# The integration of virtual reality and EEG: A step-by-step guideline

**DOI:** 10.1016/j.mex.2025.103770

**Published:** 2025-12-18

**Authors:** Caspar Krampe, Juriaan Wolfers, Philip Dean

**Affiliations:** a Marketing and Consumer Behaviour Group, Wageningen University and Research, Building No. 201 (Leeuwenborch), Hollandseweg, 1, KN Wageningen, 6706, the Netherland; b Information Technology Group, Wageningen University and Research, Building No. 201 (Leeuwenborch), Hollandseweg 1, KN Wageningen, 6706, the Netherland; c School of Psychology, University of Surrey, Guildford, Surrey, UK

**Keywords:** Virtual Reality (VR), Electroencephalogram (EEG), Data harmonisation, Neuropsychological Measurements, Guideline, Protocol

## Abstract

Virtual Reality (VR) is gaining traction in cognitive and decision-making research because of its ability to generate immersive, controlled environments that closely replicate real-world situations. Its integration with neurophysiological tools such as electroencephalography (EEG) and eye-tracking offers a unique opportunity to gain deep insights into consumer behaviour by combining behavioural and neural measures in real-time. However, the simultaneous use of VR and neurophysiological measures remains challenging due to crucial issues concerning data stream alignment, event timestamping, hardware compatibility, and potential signal interference induced by head-mounted equipment. To date, the absence of standardised protocols has limited the scalability and reproducibility of multimodal VR research, thereby hindering its widespread adoption. This paper presents a detailed, step-by-step guideline for harmonising EEG, eye-tracking, and VR data streams using the Lab Streaming Layer (LSL) in a Unity-based VR environment. A Varjo headset with in-built eye-tracking and a Neuroelectrics Enobio EEG system are used as a working case to illustrate a practical implementation of the guidelines displayed. By outlining clear guidelines for hardware configuration, event timestamping and software implementation, this paper demonstrates how open-source tools can enable high-precision data synchronisation in immersive research setting. The protocol is flexible and transferable to similar setups and therefore supports cross-study comparability and encourages wider uptake of multimodal VR methodologies, while acknowledging methodological constraints.

## Graphical abstract

**Figure:**
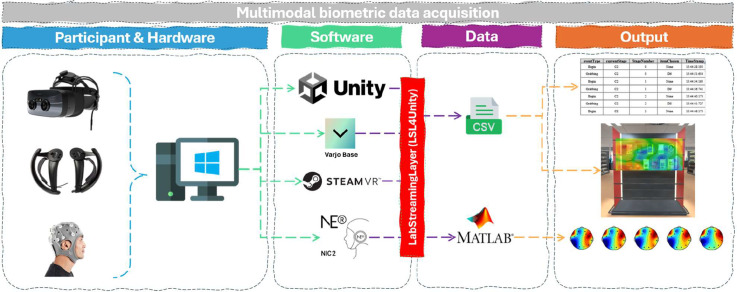

## Specifications table

**Table:** 

Subject area	Neuroscience
More specific subject area	Consumer Neuroscience; Marketing; Data Harmonisation
Name of the reviewed methodology	Virtual Reality, Eye-Tracking & EEG
Keywords	Data harmonisation, EEG, Virtual Reality, Eye-Tracking, Neuropsychological Measurements, Guideline, Protocol
Resource availability	Software: Unity, LSL, NIC, Varjo, SteamVR, MATLAB (optional)
Review question	How to harmonize the different data streams of VR and EEG?
Sample code (GitHub)	https://github.com/MCBLaboratory/Virtual-Reality

## Background

Virtual Reality (VR) is increasingly applied in cognitive and decision-making research, including marketing and consumer studies, due to its ability to create immersive yet controlled environments that simulate real-world scenarios while maintaining experimental rigour [2]. Advances in VR technology enhance its value for studying consumer behaviour, cognition, and emotional responses, especially when integrated with additional data sources.

Combining VR with neurophysiological tools such as electroencephalography (EEG) and eye-tracking enables researchers to gain deeper insights into decision-making processes. Eye-tracking in VR can generate gaze data and time-specific heatmaps that reveal how visual stimuli capture attention. EEG complements this by measuring neural activity related to cognitive and affective processing.

Despite its promise, the integration of VR with neurophysiological measurements like EEG and functional Near-Infrared Spectroscopy (fNIRS) [3] remains underdeveloped. This limits the ability to fully explore the neural mechanisms underlying decision-making in immersive settings. More precisely, one major obstacle to neuropsychological and VR data integration is the alignment of data streams, which requires addressing technical complexities such as combining data streams from different software packages to ensure accurate and consistent timing of events across these streams. In addition, it must be possible for events of interest in the VR setting to be accurately time-stamped in these data streams. Different hardware configurations must be considered as they add further complications on the type and timing of data they can support (e.g. in their sampling rate). Finally, the use of VR with other neuropsychological devices on the head (e.g., EEG or fNIRS) will affect where the sensors can collect data from, whilst ensuring participant comfort and reducing any signal noise (e.g. electrical noise in the EEG signal from the VR hardware).

Hence, the creation of standardised protocols and guidelines is required to aid uptake and usage. These will help consumer/cognitive neuroscience researchers in cross-study comparisons and reproducibility as studies are conducted to a similar standard. Without a reliable framework for data alignment, the validity of multimodal VR research remains uncertain, making the combined application of both methods inaccessible to researchers.

This paper introduces a standardised protocol for harmonising EEG, eye-tracking, and VR data streams using the Lab Streaming Layer (LSL). The guidelines use a specific setup of a Unity VR environment, Varjo VR set with in-built eye-tracking, and a Neuroelectrics Enobio EEG, but the principles detailed should also work with other setups that have the hardware and software capabilities required. Unity accounts can be created for free, and environments can be created and used in non-commercial academic research settings with some flexibility. LSL is an open-source software package which provides a robust framework for real-time data synchronisation, ensuring high temporal precision in EEG recordings. EEG was used as a working case as it requires a high temporal resolution compared to fNIRS. Hence, if data harmonisation works with EEG, it will also work with less temporal resolution measurements. We also utilise some Unity coding to time-stamp events within the VR environment, dependent on user behaviour. The protocol offers a step-by-step guideline to setting up and synchronising mobile EEG with VR, covering hardware configuration, software implementation, and programming requirements. By leveraging open-source solutions, this approach enhances accessibility, allowing researchers to integrate EEG and VR seamlessly without relying on proprietary software or complex setups.

## A step-by-step guideline

Step 1: Technical requirements

Given the computational load of running both EEG data captured experiments, eye-tracking and a VR experiment with a high-resolution VR headset simultaneously, the following requirements are recommended to ensure a smooth running of the experiment.

**Fig. 1:**
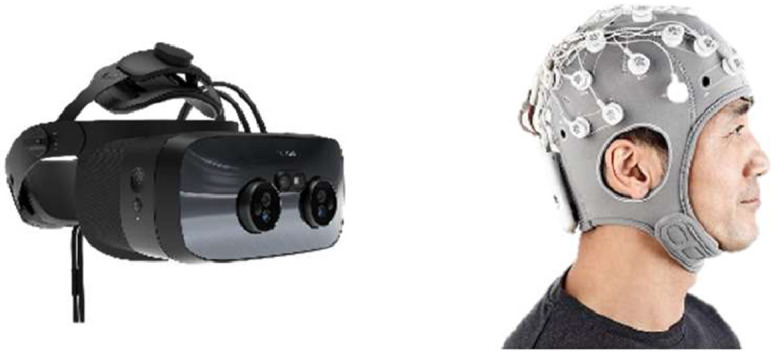
Varjo XR3 headset and Enobio’s 32 channel EEG system.

A Varjo XR3 PC VR headset was used for the experiment, utilising its eye-tracking functionality. Unity (https://unity.com/) was used to create and present the VR environment (v2022.3.28f1, with Unity Hub 3.8.0) (Fig. 1).

The Enobio 32 EEG system from Neuroelectrics was used, with EEG data recorded via Neuroelectrics Instrument Controller (NIC) software (v 2.1.3.6) (Fig. 1).

One desktop PC was used to run both the VR setup (including eye-tracking) and the EEG recording. This was an Alienware Aurora Ryzen Edition R14 PC running Windows 10 (22H2, 10.0.19045), with AMD Ryzen 9 5900 × 12-Core processor (CPU), 64GB RAM, and a NVIDIA GeForce RTX 3090 Graphics card (GPU) with 24GB VRAM.

Other PC setups may enable the same data collection if it fit the manufacturer’s hardware requirements. The following desktop PC requirements are provided by Varjo and Neuroelectrics^1^ (see Table 1).

**Table 1:** Technical hardware requirements VR & EEG headset.

	Varjo XR3	Enobio EEG 32 channels, NIC2 software
Processor (CPU)	Recommended: Premium 8-core CPU. One of the following or better: Intel Core i9–9900 K, Intel Xeon W-2245 8-core, AMD Ryzen 7 3700X Minimum: 8-core CPU, One of the following or better: Intel Core i7–9700k, AMD Ryzen 7 2700X	Processor: 1.6 GHz
Graphics Processing Unit (GPU)	Recommended: NVIDIA GeForce RTX 4070Ti, NVIDIA GeForce RTX 3080, NVIDIA RTX 6000 Ada, NVIDIA RTX A6000 Minimum: NVIDIA GeForce RTX 4070Ti, NVIDIA GeForce RTX 3070, NVIDIA GeForce RTX 2080 Ti, NVIDIA Quadro RTX 60,00^2^	-
Memory (RAM)	32 GB (DDR4 / DDR5)	2 GB
Storage space	2GB	-
Video output	2 x DisplayPort 1.4	-
USB connectivity	2 x USB-A 3.0/3.1	Interfaces: USB, WIFI and/or Bluetooth® (3.0 or 2.1)
Operating System (OS)	Windows 10 (64-bit) Windows 11	-

2 The following software requirements involve Unity for the VR environment and its corresponding packages, followed by the Varjo and Enobio software.

For the software, a separate Varjo XR subscription is required when using a Varjo headset, consisting of a monthly subscription version or a lifetime version, depending on the purchase. The EEG software, NIC2, is open source and free to use (Table 2).

Step 2: Basic VR setup

**Table 2:** Technical software requirements.

	VR environment (inc. eye tracking)		PC VR: Varjo		EEG LSL: Enobio
Required	- Existing Unity-based VR experiment - Unity version 2021.3 LTS or higher - OpenXR Plugin version 1.10.0 or higher - Varjo Unity XR SDK Plugin - Lab Streaming Layer version 1.16.0 - XR Interaction Toolkit version 2.5.3 or higher	- Desktop PC running Windows 10 or 11 that meets the (PCVR) system requirements^3^ - Varjo headset (XR-4, XR-3, VR-3, and Varjo Aero) - Varjo Base version 4.7.0 or higher			- Desktop PC running Windows 10 or 11 that meets the system requirements - (**Tested with**) Enobio 8/20/32 EEG device; Starstim 8 5 G (Firmware version 4021) - (**Tested with**) NIC2 (NE Neuroelectronics®) version 2.1.3.11 or higher
Optional		- SteamVR (OpenXR/OpenVR enabled)		- SteamVR for lighthouse-based tracking (inc. Valve Index controllers)	- SteamVR for lighthouse-based tracking - Latest Varjo Base^4^ - Varjo Unity XR SDK Plugin^5^
(Alternatives; not currently validated^6^)		Meta Quest Pro (incl. eye tracking) Meta Quest 2/3/3S Valve Index HTC VIVE Pro 1/2 (incl. eye tracking) HP Reverb G1/G2 Bigscreen Beyond 1/2 (incl. eye tracking v2)			

3 https://varjo.com/use-center/get-started/varjo-headsets/system-requirements/.

4 Depending on the VR headset manufacturer.

5 https://github.com/varjocom/VarjoUnityXRPlugin.

6 In theory, any (modern) PC VR capable headset will work with the OpenXR toolkit and the EEG LSL integration. Eye tracking will depend on the VR headset support and the available Unity-specific SDKs to incorporate it in the experiment.

This tutorial provides a guide to incorporate VR eye tracking and EEG data logging with an existing Unity-based VR experiment, utilising the *OpenXR*^2^ and *VarjoUnityXRPlugin*. An assumption is made that the project is already up and running in Unity’s Play mode, utilising Varjo’s (or any other VR headsets) inside-out or outside-in tracking capabilities^3^ in combination with a working (open) XR-rig. For additional information on setting up a working Unity project with the OpenXR-rig, please consult Unity’s OpenXR manual here. A Unity project with an OpenXR-rig, containing simple data logging and EEG capture, is provided in Section 5. The *Open XR* and *VarjoUnityXRPlugin* plugins can be added through Unity’s Package Manager (UPM) via *Unity – Window – Package Manager*. The *OpenXR* plugin is a standard plugin in the package library and can be enabled when setting up new (or existing) Unity projects. The Varjo Unity package can be accessed on their official GitHub page and added using UPM. As a final step, the Varjo XR Plugin checkbox must be checked within Unity’s *Project Setting - XR Plugin Management – Varjo*.

Each VR setup would typically include a scenario, experiment or task where participants’ behaviour is being studied, and the behavioural outcome (e.g. choice), eye-tracking data and neurophysiological data (e.g., EEG) related to that behaviour are collected. The example used in this paper is a multimodal neurophysiological consumer study to explore the first-choice brand effect (cf [4]) of sustainable packaging. Participants were placed in a virtual supermarket with various packaging designs; each altered with or without sustainability elements (e.g., logo, label, text) .^4^ To map the (neuropsychological) effects of sustainability packaging elements on participants' ‘first choice’, insights regarding participants' behavioural choices, their eye movements and their neuropsychological state (esp. in the prefrontal region) were recorded. During the task, participants are shown two product packages on a shelf and asked to choose between them by picking one up. They are shown several different packages over several trials, with breaks between, using an experimental block design. Tea packages are manipulated to highlight some differences in sustainability through labelling and images. The captured VR experiment data included a timestamp, event type indicating the tasks of the participant (e.g., “Begin” or “Grabbing”), the type of packages presented in each trial (incl. left/right positioning) and the chosen package design by the participant.

Step 3: Eye tracking setup

Varjo offers an excellent template that provides gaze data logging, calibration functionality, and more. This template (.cs) can be found on the official Git page.^5^ The output of the Varjo template generates a wide range of information, including eye location coordination and gaze vector positions. However, it does not link these eye-tracking data to any specific virtual objects or events within the VR environment. Modifications in both the VR environment and the required scripts must be made to make this data event- or object-based (i.e., eye-tracking heatmaps or fixation duration per stimuli). A collider-based implementation, where a virtual object with a collider (mesh or box, depending on the shape) attached to it was used to record when the gaze of a participant ‘collided’ with this virtual object, marking the name of the object and the time-event in a data logging file. This effectively shows the timeframe that the gaze was on the specific object, allowing task and/or object-based analysis. By strategically placing and naming this collider-based (parent) object, data collection during specific time frames on an area of interest becomes possible.

An example of capturing eye-tracking areas of interest (AOI) on participants’ buying behaviour can be found in our example experiment (see Step 2: Basic VR Setup), which aims to capture the effect of sustainability labels or images on packages (e.g. packages of tea) in a virtual supermarket. This collider-based implementation is done by strategically adding collider elements on these specific parts (e.g. sustainability labels) of the virtual packaging. It is important to assign relevant, unique and meaningful names to the package and collider element within the Unity code, as these will be used in the output data logging file, simplifying data analysis.

**Fig. 2:**
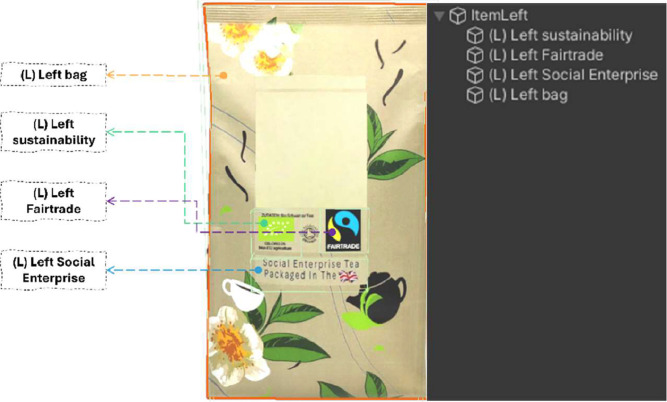
Relevant names attached to items of interest (e.g., left target tea package). The green outline indicates the Unity based box collider, triggering the data logging function (*HitObjectName*) when colliding with the gaze interactor, registering the name of the parent object (e.g., (L) Left Fairtrade).

As indicated, to ensure compatibility with the provided *EyeTrackingExample* script provided by Varjo, adjustments must be made. The gaze ray cast implementation must be modified to implement a collider-based approach. In the generated output file, a new column will be added that logs the name of the *GameObject* (the virtual object of interest) through a user-placed collider named *HitObjectName*. This collider can be assigned to an empty *GameObject* attached to the object-of-interest (e.g., tea package) with a collider – mesh or box, attached to it. The proportions can be modified to fit a certain space requirement with the included *Edit Collider* function in Unity’s Inspector window. It is important to assign a relevant name to this object (*GameObject)*, as this name will be captured in the data logging file, simplifying the data analysis in a later stage. The gaze ray cast will activate the logging of the *GameObject’s* name when it collides with the *GameObject*. The final step of initialising the script is to assign it to an (empty) *GameObject* in the hierarchy and finalise the configuration through the Inspector (Fig. 2).

The visualisation transforms require three transform objects: the fixation point, the left eye & the right eye. A gaze point indicator is also required and can be helpful to test the eye tracking capabilities, as a (permanent) indicator that follows the participant’s eye gaze within the game-view throughout the task. These objects are already attached to the OpenXR prefab provided in the Unity template of this paper. Otherwise, the Eye-tracking sample scene, located in the Varjo GitHub repository, contains these objects. Keep in mind to assign the correct objects in the Eye-tracking script via the Inspector and/or change the designated eye tracking logging key. These objects can be exported as a prefab and placed in the project of interest. The modified *EyeTrackingExample.cs* we used, can be found in the GitHub repository of this paper.

Eye tracking data, collected with a sample rate of ∼200 frames/second, consisted of multiple labels, with the *HitObjectName* and *CaptureTime* being the most relevant for synchronising^6^ with the experiment data.

With gaze data now linked to specific virtual objects using collider-based detection, providing time-stamped logging for object or event-based analysis, we can begin to explore more complex interactions by combining eye tracking with other biometric data, resulting in a multimodal biometric data acquisition. To extend this framework, the next section will provide an overview of combining VR Eye-Tracking data with EEG data logging.

Step 4: Connecting EEG and Unity data with LSL

To link the neurophysiological data collected (in this case, EEG) to the VR environment (stimuli) and/or participant behaviour, we require a method of marking specific events within the EEG data with timestamps. This must be done in real-time with high temporal resolution, precision and low variance to allow accurate and reproducible analysis of neural changes around a specific event.

For our specific setup, we had to link the participant’s experiment data, eye tracking data (as described above) and EEG data logging. Our proposed solution is to use the Lab Streaming Layer (LSL) communication protocol (https://labstreaminglayer.org), which can be used to synchronise data from multiple multimodal “streams” or devices into one dataset in real-time using “inlets” and “outlets” (see user guide: https://labstreaminglayer.readthedocs.io/index.html). In this example, LSL is utilised to stream (1) EEG data from the EEG recording software (Neuroelectrics NIC2) and (2) behavioural/experimental event information from Unity, combining them into one dataset that contains both the EEG data and the event ‘markers’ (labels) with high temporal accuracy and a specific event based on the marker added to the data. These events can be stimulus events (e.g. when an object is presented to a participant), behavioural events (e.g. when a participant touches or chooses a stimulus), experimental cues (e.g. when a task starts or ends) and interaction events (e.g. when a specific combination of stimuli & behaviour occurred). Your choice to use these would be dependent on the design and scope of the VR experiment.

For the setup described in the article, several assumptions are made: (i) that the project is already up-and-running in Unity with the OpenXR toolkit, and that there is some form of data logging^7^ on event markers; (ii) that the EEG headset is successfully capturing (manually added) event markers when being run in isolation outside the Unity environment (i.e. it is functioning correctly and setup to receive markers); (iii) that the Neuroelectrics Enobio EEG headset and recording software (NIC2) work in this setup with any PC-related VR headset that is compatible with the OpenXR toolkit. We chose Neuroelectrics due to it being the EEG setup within our labs that enables wireless or Bluetooth data streaming, has a relatively quick setup, and modification of placement of electrodes are possible when necessary. Other EEG headsets and software are likely to work with this setup, but further testing would be needed to confirm - please check their compatibility with LSL and the other components used.

Lab Streaming Layer has various plugins to allow it to stream data from different software and setups.^8^ The plugin for Unity is called LSL4Unity and was developed by @xfleckx. The package can be added within Unity in the *Window - Package Manager – add package from git URL*.^9^ In this example, event ‘outlets’ will be used to submit event data (e.g., grabbing product X [fair trade tea package] during stage Y [trial 2, Block 2]) from Unity and then link this with the ‘inlet’ of the data stream from the EEG (Neuroelectrics NIC2 software) to create a data set with both the event data and the EEG data temporally aligned. This example was also set up where this event data was marked through variables, followed by submitting this event data to a .csv file afterwards to enable data quality checks and corrections. This approach is recommended, if possible, within the experiment design. However, this may depend on the nature of data logging for your project

The task in this example involved the previously mentioned example scenario in step 2: Basic VR Design. Upon each designated ‘event’ participant interaction (in this case, when participants selected a package by grabbing it) data was sent to (1) a *.csv* file for the experiment data logging (this is the VR experiment data mentioned previously [timestamp, event type, stage number, the type of packages presented in each trial (inc. left/right positioning) and the selected package]), (2) a *.csv* file for the eye tracking data (inc. capturing the experimental data, this is also mentioned previously [collected at 200 frames / second, consisting of multiple labels, with *HitObjectName* and *CaptureTime* being the most relevant for synchronising with experiment data]) and 3) a *.mat* file (MATLAB) with the EEG headset through NIC2, capturing both EEG - and the corresponding experiment data.

Data logging of the experiment and EEG data was initiated by the *OnObjectGrabbed* function from the OpenXR Interaction Toolkit. This function activated the recording scripts whenever a participant selected a package, either by squeezing or pressing the Valve Index controller trigger, simultaneously submitting the event data from the experiment (*timestamp, event type etc*.) into the EEG data stream through LSL. The LSL sample included a predefined identifier (e.g., ‘*FavoriteProductGrabbing*’), followed by the content of the experiment data (*timestamp, event type, item packaging options etc.*) formatted as a string. Both the identifier and the data structure can be customised and/or extended depending on the scope and design of the VR experiment.

EEG data was recorded with Neuroelectrics NIC 2 (v2.1.3.6) using a WiFi connection from 8 electrodes positioned over the pre-frontal and central cortex (Fp1, Fp2, Fpz, AF7, AF8, C3, C4, Cz), capturing raw EEG voltage data (in µV) per electrode at a sampling rate of 500 Hz. Ground (DRL: Driven Right Leg) and Reference (CMS: Common Mode Sense) were positioned behind the left ear on the mastoid bone.^10^ These ground and reference electrodes were attached using Kendall H124SG single-use electrodes. The scalp electrodes were setup using Signagel. Typical EEG procedures were applied (cap size chosen to best fit participant, centralise Cz relative to nasion/inion and tops of ears and then start setting up the EEG electrodes with gel for good signal quality.

**Fig. 3:**
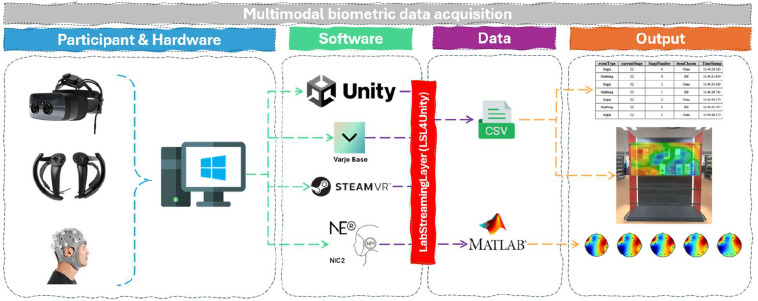
Multimodal biometric data acquisition.

To modify the data logging script to include sending the event markers to LSL (after ensuring LSL4Unity is loaded in the package manager), please follow the included EEG LSL example script from the GitHub repository of this paper (Fig. 3).

Step 5: Data Collection and Validation

For the setup of the hardware and software, it proved important to follow a specific sequence. In this case, all the software (Unity, LSL, NIC2 for EEG) is running and executed on the same PC, which is turned on and logged in.

1) The first part of the setup is to place the EEG device and check the signal quality. Once all the electrodes were arranged with good signal quality, the EEG amplifier is disconnected to allow the VR headset to be attached.
2) The VR headset is prepared. The Unity experiment project and Varjo Base are initialised, and the base stations are powered on. SteamVR is then started, utilising outside-in tracking with the Valve Index controllers^11^ and base stations. The Varjo headset is placed on the participant’s head, which automatically triggered the eye calibration process and launches SteamVR tracking.
3) The EEG amplifier is then re-connected and linked with NIC2 again to check the signal quality, and that data is being sent OK. Afterwards, LSL is opened on the PC and set up to receive event data from the VR environment and EEG data from NIC2 and Neuroelectrics. Data recording from the EEG is started.
4) After the eye-tracking calibration process, the researcher activates Unity’s play mode, which starts the VR experiment, begins data logging and establishes a connection with the NIC2 software via the LSL. To enable eye tracking, the researcher needs to press the designated key (F2 is the default key; configurable in the Eye-Tracking script) to start the eye tracking logging.
5) At the end of the experiment, the researcher stops the EEG recording in NIC2 and removes the VR headset before stopping Unity’s play button to avoid potential in-game stutters. LSL should be stopped as the last step in the data collection procedure.

## Challenges, limitations and conclusive remark

While the integration of VR and EEG technologies demonstrates promising potential for immersive neurophysiological research, several challenges remain and must be acknowledged.

The current setup has only been tested using event-based stimuli. Although the system supports broader functionalities, such as triggering experimental cues or streaming other types of experimental data to the EEG software, these have not yet been implemented or validated. Extending the current framework to include a wider range of experimental protocols remains an area for future work.

The system architecture is designed for extensibility. In principle, any VR headset compatible with the OpenXR toolkit could be used. Similarly, the use of the Lab Streaming Layer (LSL) in Unity means that a variety of EEG systems beyond NIC2 can be supported. However, practical testing and validation of such configurations have not yet been carried out, and therefore interoperability across different hardware ecosystems is not guaranteed.

Specific attention should also be given to the system's end-to-end latency^12^ that has not been empirically measured. Accurate timing is fundamental in EEG research, especially when millisecond precision is required. Latency may therefore be introduced at several stages: the wireless communication between the VR controllers and the PC, the transmission of EEG signals from the amplifier to the PC (potentially wireless), and the processing delay before data reaches NIC2 or equivalent software. Table 3 provides an illustrative latency table to offer quantitative expectations of the end-to-end system latency.

**Table 3:** Illustrative end-to-end system latency.

Latency component	Example	Expected latency (ms)	Source / Notes
User-input lag	Valve Index controllers, HMD tracking, eye tracking sensor	13–58	Controller-to-screen latency [7]
Application-dependent processing lag	Unity, LSL4Unity, NIC2	∼11	Framerate physics/logic at 90 Hz XR3
Rendering lag	Scene rendering, display persistence	0.5–2	Scene dependent + manufacturer spec [7]
Synchronization lag	GPU buffering (VSync)	0–11+(frame-based)	Occurs during framerate drops below 90 Hz (HMD dependent)
EEG acquisition/transport lag	Enobio → NECBOX → NIC2 (WiFi/USB)	Variable (2–10)	Timestamp corrected, network/USB overhead (single digits)
Aggregated End-to-End System Latency	Visual event → EEG + event timestamp	∼30–90	Includes Hardware + Software latency
Human latency	Processing & reaction time	150–300	Human reaction time (HRT) visual stimuli [1]

**Fig. 4:**
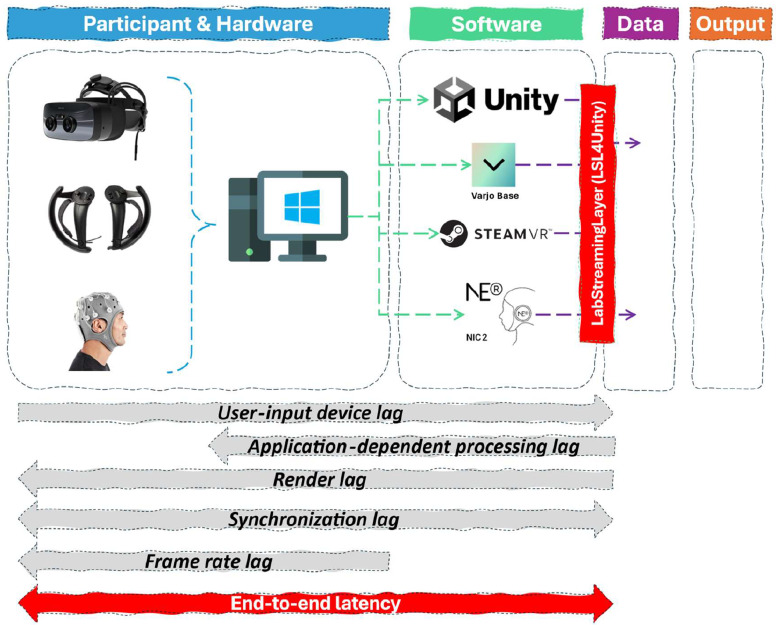
End-to-end system latency pipeline.

Accurate estimations of the system’s end-to-end latency requires proper calibration and testing of the equipment which has not been the primary focus of this paper. Nevertheless, understanding and estimating the end-to-end latency of the equipment is a crucial step towards accurate data collection and validation [5].

(System) latency can generally be divided into five main elements: (1) user-input device lag, (2) application-dependent processing lag, (3) rendering lag, (4) synchronisation lag and (5) user perceived framerate induced lag [6]. Fig. 4 provides an overview of the induced latency per stage in the equipment pipeline.

(1) User-input lag consists of the latency induced between the communication of the tracking or input hardware (e.g., Valve Index VR controllers, HMD tracking modules) and sensor-based measurements (e.g., eye tracking) and the moment the signal is registered by the software application. Typically, the delay of the Valve Index controllers between physical movement and the time it takes before the picture is shown in the HMD (controller-to-screen latency) is between 25 and 58 ms but can be reduced to below 13 ms with motion prediction algorithms in SteamVR, according to a study by Warburton et al [7] .^13^ Additional overhead occurs from wireless transmission delay and USB communication overhead of the SteamVR tracking dongles. USB overhead, however, typically consists of low single-digits milliseconds of latency.
(2) The application-dependent processing lag (ADPL) refers to the delay between the initialisation of a rendering or processing tasks (for example, the Unity game engine or NIC2) and the final computation values used for visualisation or event generation. According to Warburton et al [7], the previously mentioned tracking data are generally processed with values from a previous frame for physics or logic (including LSL4Unity) integration tasks. This results in a latency within the Unity game engine of approximately 1/90 frames^14^ or 11.11 ms. The additional latency overhead of the logic involved in Unity’s integration of the LSL4Unity package with the NIC2 software is, according to Neuroelectrics synchronised and drift-corrected through the NECBOX (Neuroelectronics Control Box) internal clock running at 500 Hz or 2 ms, integrating Unity and EEG data through proper timestamped submissions, effectively correcting for potential ADPL. It should be noted that the EEG data during the experiments was recorded via Wi-Fi rather than a wired USB connection. While the transmission control protocol (TCP) ensures no data packages are lost, external factors such as the traffic of the network or the distance from the NECBOX to the router make it difficult to quantify the latency. Future research should consider using a wired USB connection to minimise and account for the induced latency between the EEG headset and the PC.
(3) Rendering lag refers to the time required to generate each frame and varies depending on the scene complexity, lighting, physics and viewpoint during the application runtime. In most cases, the display persistence of a VR HMD is between 0.5 and 2 ms and occurs after the ADPL stage [7].
(4) Synchronisation lag represents the total time that data is delayed between the processing stages of the rendering pipeline. The application is still processing previous data, resulting in temporarily queuing the influx of new data. An example is Vertical synchronisation (VSync), where a delay occurs when the graphics card synchronises with the display's frame rate. Although VSync can reduce potential tearing of the image, resulting in a smoother image, the technique does add additional latency of 1/90 frames or 11.11 ms through the GPU’s back – and front buffer when it cannot hit the required framerate. As a result, frame-rate induced lag happens when the data displayed becomes out-of-sync. Technically speaking, framerate-induced lag is not considered part of end-to-end latency but is perceived by the user. VR experiments must consider proper optimisation of the experience that aims to run at the required refresh rate of the HMD.
(5) Finally, fluctuating human reaction time and its participant-dependent processing time of both audio – and visual stimuli should also be considered when incorporating EEG data with a VR experiment. Repetitive VR tasks, as usually done in neuropsychological experiments, could be a solution, allowing comparison against a participant-specific baseline.

Hence, when analyzing the EEG and event data, it remains important to apply an offset to the event timestamps to account for the total end-to-end latency. For example, if a participant sees two new stimuli at 01:00:000, you should adjust the EEG event markers by adding the measured system latency (e.g., +90 ms), resulting in a corrected reference time of 01:00:090 and so forth. In addition, you may also factor in the estimated human response latency (approximately +250 ms) for more accurate temporal alignment between stimulus presentation and neural activity.

A further set of limitations involves data quality and signal integrity. VR headsets are known to introduce significant electrical noise, which can contaminate EEG recordings. Additionally, motion artefacts, caused by head, body, or eye movements, as well as by cable motion or headset pressure on the EEG cap, pose a substantial challenge. While these factors are more controllable than in real-world settings, they still limit the precision of signal interpretation compared to traditional lab-based experiments. Placement constraints for EEG electrodes, especially when accommodating a VR headset, further reduce the flexibility of data acquisition. These issues may be even more pronounced when using modalities like fNIRS, due to their larger sensor size and greater sensitivity to movement-induced noise.

We present some data from our setup in the supplementary material to allow researchers to see how the data was affected in our case (power for electrical noise (49–51 Hz to cover 50 Hz peak) and signal drift (0.1–1 Hz to cover artefacts of sweat and some movement artefacts, and illustration of raw data from two participants). Typical frequency ranges for analysis during tasks (theta, alpha, beta; approx. 4–30 Hz) may be less affected by the peaks at 50 Hz and low frequency drifts (see Supplementary Material), but the data illustrate the importance of good EEG setup (impedance, ground), design and cleaning process when using this setup. Important aspects that need to be considered in the study conceptualisation but are not yet in the experimental design phase.

Task design also presents challenges. While the VR environment offers more ecological validity than lab tasks and more control than real-world observation, designing reproducible and precisely timed stimuli in VR still requires careful planning. The trade-off between immersion and experimental control remains a methodological consideration.

Finally, participant-related factors must be noted. VR-EEG setups generally require longer setup times, which may lead to participant fatigue, dropout, or sampling biases. However, the extensibility of the system may also enable the integration of additional physiological signals, such as heart rate, skin conductance, and respiration, opening avenues for broader psychophysiological research, albeit at the cost of added complexity.

To conclude, while the proposed VR-EEG integration is highly flexible and theoretically scalable, its current implementation requires additional testing, as it is affected by noise and latency issues that require technical proficiency and careful task design. Addressing these limitations through further validation, latency benchmarking, and signal artefact mitigation strategies will be essential to realise its full research potential.

From a technical perspective, the primary prerequisite is a working Unity-based VR environment that includes data logging functionality. While the implementation steps are relatively minor for experienced developers, this still introduces a barrier to adoption for non-technical researchers. We hope that these guidelines and the provided GitHub repository also help less experienced developers/researchers to harmonise complex VR data streams.

## CRediT author statement

**Caspar Krampe**: Conceptualisation, Data Curation, Funding Aquisition, Investigation, Methodology, Project Administration Writing- Original draft . **Juriaan Wolfers**: Resources, Software, Visualisation, Methodology, Writing- Original draft preparation. **Philip Dean**: Conceptualisation, Methodology, Data Curation, Formal Analysis, Methodology , Writing- Original draft preparation.

## Declaration of competing interest

Please tick the appropriate statement below (please do not delete either statement) and declare any financial interests/personal relationships which may affect your work in the box below.

The authors declare that they have no known competing financial interests or personal relationships that could have appeared to influence the work reported in this paper.

## Appendix Supplementary materials

Image, application 1

## Footnotes

11 SteamVR for the device setting in Varjo Base was selected instead of XR / inside-out tracking. Depending on the headset, any compatible VR controller that is compatible with the OpenXR toolkit will work (e.g., Oculus Touch controllers). In the case of the Varjo XR3 – no controllers are included by default, but can be added afterwards.

13 The latency of the controllers has been tested with a Valve Index HMD and not the Varjo XR3, which makes it not a like to like comparison of the screen latency.

14 Following Varjo’s recommended 90 frames per second, and no additional framerate induced latency and / or VSync or motion smoothing enabled.

## Footnotes

1 Several other OpenXR compatible VR headsets can be used as well depending on the VR experiment and packages used, (potentially) lowering the system requirements. Increasing VR complexity (complicated VR scene, higher fidelity graphics etc.) or the EEG headset (more EEG channels, higher sampling rate etc.) may require an increase of (processing) power to ensure no data loss or performance issues. Please see: https://github.com/MCBLaboratory/Virtual-Reality.

2 Royalty-free open standard for developing XR applications for a wide array of AR and VR ecosystems and devices.

3 This method has been tested with a Varjo XR3 headset that utilized three external SteamVR lighthouse base stations V2 (outside-in tracking) and Valve Index controllers.

4 Ethical approval was granted by the ethics committee of the institutions involved in this study. Each participant gave informed consent to the studies that trialed this technique. Future studies, following these guidelines, should ensure independent ethical evaluations before starting data collection.

5 Samples~/HDRP/EyeTracking/Scripts/EyeTrackingExample.cs.

6 The experiment data was captured independently of the eye tracking data, requiring manual harmonization of the data with the captured timestamp of each event.

7 A data logging method is to record contents of state variables in a .csv file when a certain object or event is changed in-game. In the case of the example experiment, the OnObjectGrabbed(XRBaseInteractor interactor) of tea packages method triggered an event where information of the stage number, type of packages and event type were recorded in a .csv file.

8 https://labstreaminglayer.readthedocs.io/info/supported_devices.html.

9 https://github.com/labstreaminglayer/LSL4Unity.git.

10 https://www.neuroelectrics.com/wiki/index.php/Neuroelectrics'_Wiki.

12 Time difference between user input and system response [6].
