# Supplementary material for: The integration of virtual reality and EEG: A step-by-step guideline
Source: MethodsX. 2025 Dec 18;16:103770. doi: 10.1016/j.mex.2025.103770 (PMC12808493; doi:10.1016/j.mex.2025.103770)
Supplement: Supplementary file 1 [file mmc1.docx]

**Supplementary Material**

EEG Data from VR EEG Setup

*Methods*

A small sample (N=3) of participant data is shown here, analysed with BrainVision Recorder 2.2.0.7383.

A Fast Fourier Transform (FFT, Power [µV^2^] normalised to 1-40Hz range, Full spectrum, Hanning Window [Periodic, Length 10%]) was performed on:

1. raw segmented data (segmented into 2s segments, overlapping by 0.5s)
2. filtered segmented data (Zero phase shift Butterworth filters, low cutoff: 0.1Hz, high cutoff: 40Hz; segmented into 2s segments, overlapping by 0.5s)

After this, the segments were averaged to make a) pre-filter average and b) post-filter average.

Finally, the power was exported (as mean value of spectral line) for a) 49-51Hz (for electrical noise) and 0.1-1Hz (as indication of ‘drift’ or noise related to no sweat or some aspects of cap/electrode movement.

This output was then a normalised value (%) of power (µV^2^) compared to the average power in the frequency of 1-40Hz. In this case, then 100 would mean the same power level as that in 1-40Hz, more than 100 means greater power and less than 100 means less power. This enables us to see any issues with electrical noise and drift, and how this changes after cleaning.

In addition to this, for visual purposes, we show the raw data and filtered data for one participant, along with pre- and post-eye artefact corrected (using Ocular Correction ICA with Fp1 as VEOG, slope algorithm for blink detection and 500s segment used for ICA, where the component was then semi-automatically removed).

Finally, for completeness, we did artefact rejection on this participant to show the number of removed segments indicating data quality (max. allowed voltage step: 50 µV/ms; max. allowed difference of values in intervals: 200 µV in 200ms; y: Lowest allowed activity in intervals: 0.5 µV in 100ms).

*Results*

Pre-filtering electrical noise was 19.96±18.89 [Mean±SEM] for Fpz, and 10.41±4.09 for Cz, this dropped post-filtering to 7.94±7.45 (Fpz) and 4.76±1.88 (Cz).

Pre-filtering low frequency drift was 102.70±19.72 for Fpz, and 180.20±77.64 for Cz, this dropped post-filtering to 89.67±15.69 (Fpz) and 159.05±49.45 (Cz).

As the sample was small, and this is pilot data, there was a large variation in noise per participant (e.g. one participant had electrical noise at 57.7 pre-filter for Fpz (Figure S1), and another had low frequency at 314.5 for Cz, whereas another had lower values for both (0.5 and 45, respectively, Figure S2). This illustrates the potential variations in setup leading to differing noise that need to be accounted for.

The percentage of segments rejected during artefact correction across participants was 11.31±3.39, or approximately 57 segments out of 500 as a proportion.

Figure S3 shows the average normalised frequency for Fpz across all participants for raw data (A) and filtered (B).

Figures S1 and S2 also illustrate blinks and eye movement in the data (A & B) and subsequent correction (C).


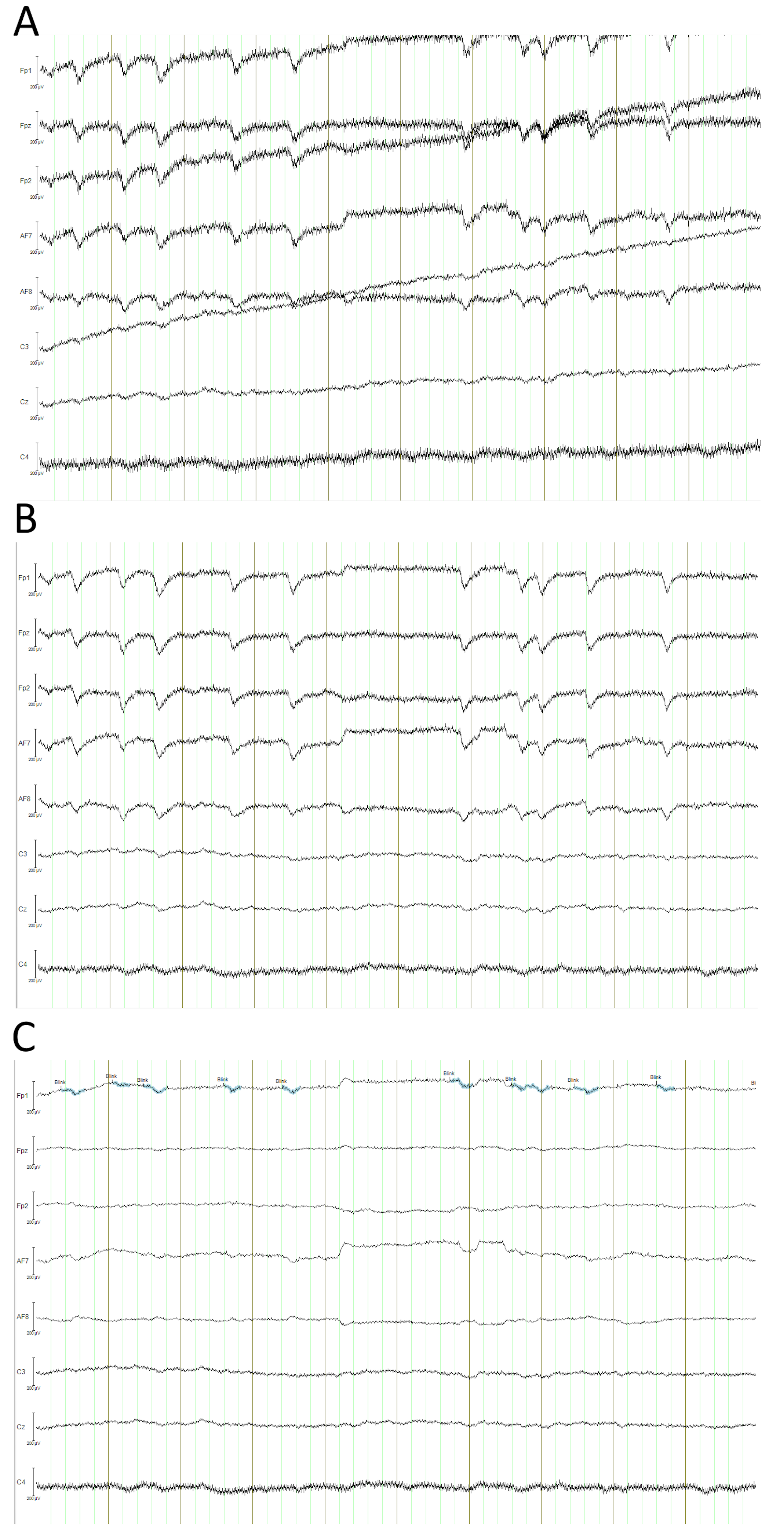


**Figure S1**. EEG Trace for participant 1. 8 Electrodes, Fp1, Foz, Fp2, AF7, AF8, C3, Cz, C4. Over 10s of data at 200µV scale. **A.** Raw data, with illustrated electrical noise, low-frequency drift and blinks. **B**. Filtered (0.1-40Hz) data for the same time point. **C.** Data after blink correction for the same time point.


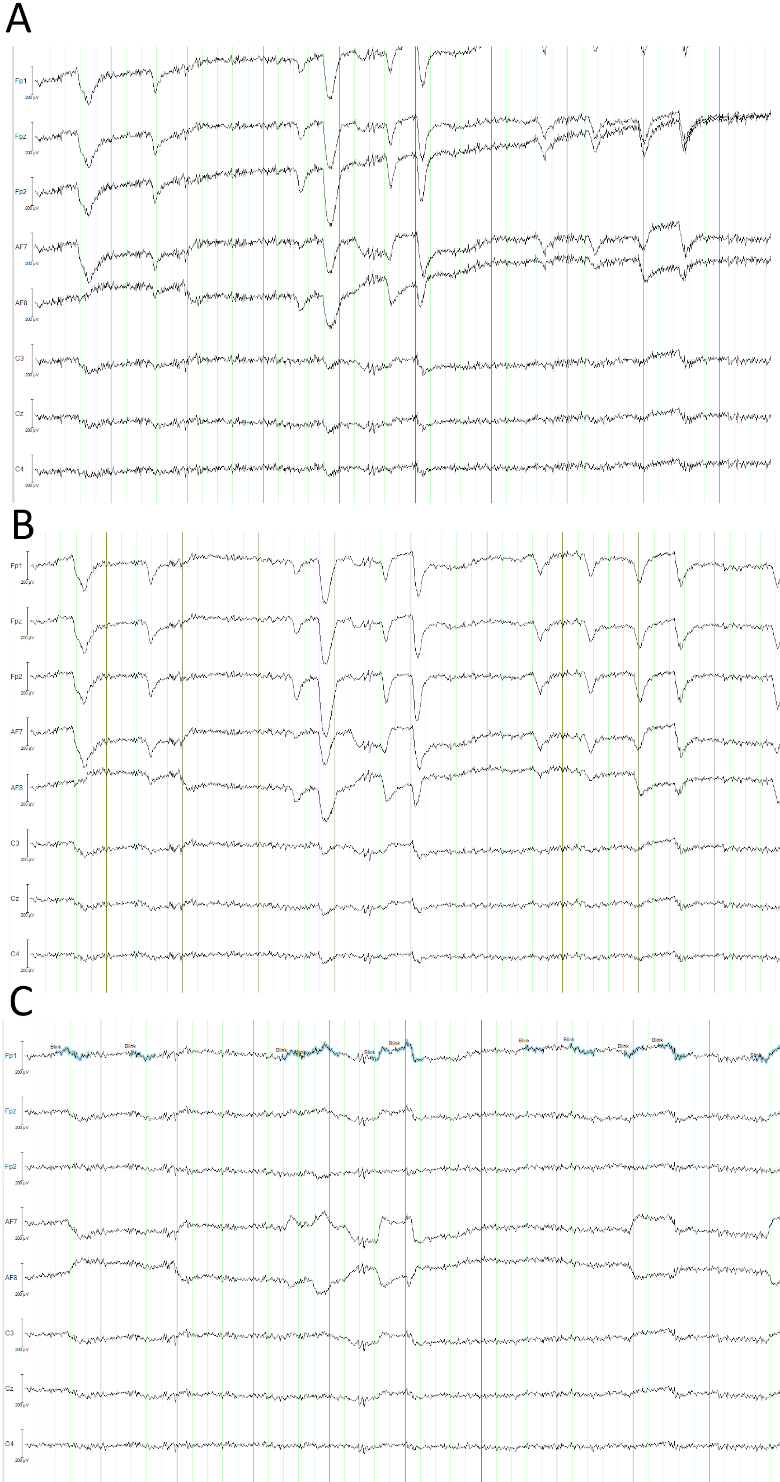


**Figure S2**. EEG Trace for participant 2. 8 Electrodes, Fp1, Foz, Fp2, AF7, AF8, C3, Cz, C4. Over 10s of data at 200µV scale. **A.** Raw data, with illustrated electrical noise, low frequency drift, eye movements and blinks. **B**. Filtered (0.1-40Hz) data for the same time point. **C.** Data after blink correction for same time point.


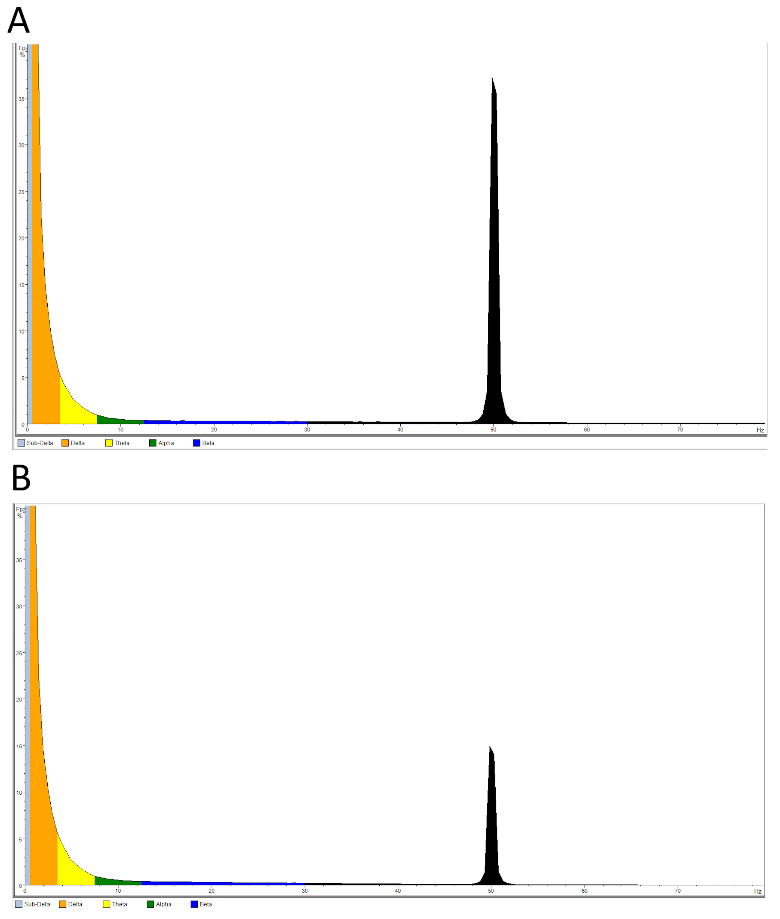


**Figure S3**. Fast Fourier Transform averaged across all 3 participants. Showing electrode Fpz. X axis: from 0-80Hz, with y-axis scale up to 40% normalised power. **A.** Raw data. **B.** Filtered (0.1-40Hz) data
